# Mr. Simeon Snell

**Published:** 1909-04-24

**Authors:** 


Mr. Simeon Snell, F.R.C.S., of Sheffield, President of the British Medical Association, and one of the leading ophthalmic surgeons of the North of England, died on Saturday, April 17, at his residence, Moor Lodge, Sheffield. He received his professional training at Guy's Hospital and Moorfields Ophthalmic Hospital. He obtained the M.R.C.S.Eng. in 1872, L.R.C.P. in 1873, and F.R.C.S.Edin. in 1892, while last year on the occasion of the visit of the British Medical Association he received from the Sheffield University the honorary degree of doctor of medicine, and for many years was ophthalmic surgeon to the Sheffield Royal Infirmary and Blind Institution. He was Professor of Ophthalmology at the Sheffield University and was a member of the University Council. He had been President of the Ophthalmological Society of the United Kingdom and President and Secretary of the local Medico-Chirurgical Society. Last year he received the Middlemore prize of the British Medical Association for his contributions to the science of ophthalmology. For many years he was Hon. Secretary of the Sheffield Literary and Philosophical Society, and at one time he edited the *Medical Quarterly Journal.*

